# 24-Hour efficacy of single primary selective laser trabeculoplasty versus latanoprost eye drops for Naïve primary open-angle glaucoma and ocular hypertension patients

**DOI:** 10.1038/s41598-023-38550-7

**Published:** 2023-07-27

**Authors:** Yipeng Shi, Yan Zhang, Wenying Sun, Alex S. Huang, Shuang Chen, Lixia Zhang, Wei Wang, Like Xie, Xiaobin Xie

**Affiliations:** 1 Eye Hospital, China Academy of Chinese Medical Sciences, 33 Lugu Road, Shijingshan District, Beijing, 100040 China; 2 Hamilton Glaucoma Center, The Viterbi Family Department of Ophthalmology, Shiley Eye Institute, University of California, San Diego, CA USA; 3 China Academy of Chinese Medical Sciences, Beijing, China

**Keywords:** Eye diseases, Glaucoma

## Abstract

This prospective, observer-masked, randomized clinical trial was conducted between December 2018 and June 2021 at Eye Hospital, China Academy of Chinese Medical Sciences. A total of 45 glaucoma patients from Beijing, China, were enrolled in this clinical trial to compare the short-term efficacy of primary single-selective laser trabeculoplasty (SLT) to 0.005% latanoprost eye drops for the treatment of 24-h intraocular pressure (IOP) in patients with newly diagnosed primary open angle glaucoma (POAG) and ocular hypertension (OHT). Both SLT and latanoprost significantly decreased mean 24-h IOP and peak IOP, although the latanoprost group effect was more potent when compared to the SLT group (both *P*s < 0.05). Compared with the SLT group, the latanoprost group had a significant and stable decrease in IOP after treatment. The latanoprost group had a more pronounced reduction in IOP at weeks 4 and 12 (*P* < 0.05) but had no difference at week 1 (*P* = 0.097). As a first-line treatment, both SLT and latanoprost eye drops are effective in newly diagnosed POAG and OHT patients. However, the latanoprost eye drops may be better in decreasing mean and peak 24-h IOP and thus controlling 24-h IOP fluctuation compared to SLT.

## Introduction

Glaucoma is the most common cause of irreversible blindness and the second most common cause of irreversible moderate and severe vision impairment worldwide^1^. Primary open-angle glaucoma (POAG) is the most common type, with a prevalence of 0.5–3.9% in Asia^2^. In China, there were 13.12 million people with glaucoma in 2015 (accounting for 2.58% of the total Chinese population), and the number is expected to increase to 25.16 million by 2050^3^. Intraocular pressure (IOP) is the only modifiable risk factor for glaucoma onset and progression. IOP normally has a circadian rhythm (varying through 24 h), and previous studies have suggested that elevated IOP^4,5^, peak IOP^6,7^, and IOP fluctuation^8–11^ are possibly more specific risk factors for glaucomatous development and progression.

The choice of initial IOP-reducing therapy is essential for affecting the long-term outcome of glaucoma treatment. Prostaglandin analogue (PGA) monotherapy is often utilized as an initial treatment choice for POAG and ocular hypertension (OHT) because it is the most effective class of IOP-lowering drugs^12^. Meanwhile, selective laser trabeculoplasty (SLT) of the trabecular meshwork (TM) has been recently recommended as a safe and effective first-line treatment for POAG or OHT patients because of comparable IOP reduction, less ocular side effects, and the advantage of a single treatment that may mitigate the need for life-long compliant drop use by the patient^13–15^. Recently, the British National Institute for Health and Care Excellence (NICE) updated guidelines recognizing SLT as a first-line treatment option for patients with newly diagnosed POAG or OHT. However, the majority of participants were Caucasian in those studies. Additionally, the general limitation of SLT research is that most SLT studies evaluate patients during office hours (less is known about 24-h and nighttime efficacy^16^). Only a few studies have directly compared the effects of SLT to PGA drops as initial glaucoma treatment^17–20^, and even less research has been done regarding a direct 24-h efficacy comparison^21^.

Therefore, in this study, we perform a prospective comparison between SLT and 0.005% latanoprost eye drops as first-line therapy in treatment-naïve POAG and OHT patients in a Chinese population. We hypothesize that primary 360° SLT will demonstrate a comparable 24-h IOP-lowering effect using latanoprost eye drops monotherapy as initial IOP-reducing therapy.

## Methods

### Study design

This single-center, prospective, randomized clinical trial was conducted between December 2018 and June 2021. All measurements (visual field, optic disc imaging, and IOP) were made by research team members masked to treatment allocation. Clinicians and patients were not masked in treatment allocation. Using a web-based system, we randomly assigned patients (1:1) to either SLT or 0.005% latanoprost as first-line treatment (www.sealedenvelope.com). This trial was approved by the ethical committees of the Eye Hospital, China Academy of Chinese Medical Sciences. All participants provided informed consent before study enrollment. The study was registered at www.chictr.org.cn (registration number ChiCTR2200056850) and complied with the principles outlined in the Declaration of Helsinki^22^.

### Participants

Patients seen at the glaucoma department of the Eye Hospital, China Academy of Chinese Medical Sciences, were screened for eligibility. POAG was defined as angles open on the gonioscopy and optic disc or retinal nerve fiber layer (RNFL) structural abnormalities corresponding to visual field (VF) loss without secondary causes. OHT was defined as an IOP of more than 21 mm Hg without glaucomatous optic nerve structural and functional changes. POAG was classified as high-tension glaucoma (HTG) and normal-tension glaucoma (NTG). Both types exhibited open angles, but HTG was characterized by an IOP higher than 21 mm Hg without treatment, while NTG had an IOP lower than 21 mm Hg on multiple clinic visits or peak measurements before treatment^23^. Glaucoma severity stratification (mild, moderate, or severe) was determined using visual field mean deviation (VF MD) at baseline, according to Mills et al.^24^. The values of VF MD in mild, moderate, and severe POAG were more than − 6 decibels (dB), − 6 to − 12 dB, and lower than − 12 dB, respectively. Inclusion and exclusion criteria were adopted to improve diagnosis accuracy and reduce potential bias induced by confounding factors. Inclusion criteria included: (a) patients newly diagnosed with OHT, mild POAG, or moderate POAG; (b) VF MD better than − 12 dB in the study eye; (c) age ≥ 18 years; (d) no history of glaucoma medications use in either eye for ≥ four weeks; and (e) willingness to undergo SLT versus latanoprost eye drops treatment. Exclusion criteria included: (a) VF MD worse than − 12 dB in the study eye; (b) patients with narrow angles or secondary glaucoma; (c) those with very high IOP who need immediate treatment (IOP > 30 mm Hg, initially or after washout); (d) any ocular condition precluding visualization of the TM; (e) previous anterior segment surgery or glaucoma laser; and (f) pregnancy. When both of a patient's eyes met the inclusion and exclusion criteria, they were given either SLT or latanoprost therapy. However, only the data from the right eye was considered for analysis^25^.

### Procedures

Ophthalmic and medical history were obtained in all patients. Visual acuity testing, IOP measurements over 24 h, automated VF testing [Humphrey Field Analyzer (Carl Zeiss Meditec, Inc., Dublin, CA, USA) SITA Standard 24-2], gonioscopy, slit lamp examination, optical coherence tomography (OCT), optic disc imaging (Model 5000, Carl Zeiss Meditec, Inc., Dublin, CA, USA), refractive error assessment, central corneal thickness (CCT) determination, and fundus imaging were acquired at baseline. To analyze the 24-h IOP curves, the patients were hospitalized in the morning (~ 9 am). IOP was acquired at 7:00, 10:00, 14:00, 18:00, 22:00, 2:00 and 5:00. At each time point, IOP was measured three times in the sitting position with a calibrated Goldmann applanation tonometer (GAT). The mean value was used for analysis in this study. For nocturnal IOP measurements, patients were gently awakened and asked to walk 5–30 m for the examination. One certified ophthalmic technician (W.W.), masked to the treatment regimens, performed all IOP measurements using the same tonometer on each patient at all visits. Eye-specific target IOP was set for each subject based on a percentage reduction from the untreated baseline IOP and an absolute threshold according to the Asia Pacific Glaucoma Guidelines^26^ and the Canadian Target IOP Workshop^27^. The target IOP was set as follows: IOP reduction of more than 20% from baseline and a value less than 25 mm Hg for OHT; IOP reduction of more than 20% from baseline and a value less than 21 mm Hg for mild POAG; IOP reduction more than 30% from baseline and a value less than 18 mm Hg for moderate POAG. The TM pigmentation was graded: 0 = none, 1 = light, 2 = medium, 3 = dark brown, and 4 = almost black based on the Scheie's system of grading angle pigmentation^28^. After completing the baseline assessment and enrollment, participants were randomized 1:1 to the SLT treatment arm or the latanoprost treatment arm. The SLT group received a single standardized SLT treatment via a frequency-doubled Q-switched Nd: YAG laser (Ellex Medical Lasers Ltd, Adelaide, Australia) with a spot size of 400 microns and a 3-ns pulse duration. The laser energy started at 0.6 mJ and increased by 0.1 mJ until “champagne bubbles” were observed. Approximately 100 non-overlapping laser spots (25 per quadrant) were applied to cover 360° of the TM using a Latina SLT Gonio laser lens with a methylcellulose coupling agent by a single glaucoma specialist (X.X.). Following the SLT procedure, patients were not prescribed non-steroidal anti-inflammatory drugs or steroid therapy during their subsequent visits. Any complications that resulted from SLT treatment, such as inflammation and IOP spikes, were documented. An increase of more than 2 mm Hg above the average IOP values prior to treatment was considered as an IOP spike, as recorded one hour after the SLT procedure^29^. The latanoprost group received 0.005% latanoprost eye drops (Xalatan, Pfizer, New York, USA) once daily (every evening at 9 pm). Follow-up evaluations were performed on weeks 1, 4, and 12 post-laser or after the first latanoprost dose. 24-h IOP acquisition was performed at each visit using the same methods as at baseline. At each visit, patients were asked to report any adverse reactions to the treatment, the vision was measured, and slit lamp examination was performed. At 12 weeks, patients also underwent VF testing, optic nerve OCT, and fundus imaging. If the target IOP was not reached, a stepped regime of increasing topical medications was followed, including the addition of beta-adrenergic antagonists, carbonic anhydrase inhibitors, or alpha agonists.

### Outcomes

The main outcomes used to evaluate the effects of controlling 24-h IOP were: (a) mean 24-h IOP (the average IOP over the entire 24-h period for each visit) at baseline and 1, 4 and 12 weeks after treatment initiation; (b) peak IOP (the highest IOP over the entire 24-h period for each visit) at baseline and 1, 4, and 12 weeks after treatment initiation. (c) 24-h IOP fluctuation (the peak IOP minus the trough IOP over the entire 24-h period for each visit) at baseline and 1, 4, and 12 weeks after treatment initiation. (d) IOP reduction from baseline at 1, 4, and 12 weeks after treatment initiation, including 24-h reduction (the post-treatment mean 24-h IOP at each follow-up time point minus the baseline mean 24-h IOP), diurnal reduction (the post-treatment mean IOP at 7:00, 10:00, 14:00, and 18:00 for each visit minus the baseline mean IOP at the same times), and nocturnal reduction (the post-treatment mean IOP at 22:00, 2:00, and 5:00 for each visit minus the baseline mean IOP at the same times).

### Statistical analysis

All statistical analyses were performed using SPSS (SPSS for Windows, V. 26.0). Parameters with the data that followed a normal distribution were presented as standard ± deviation. Parameters with a skewed distribution of their data were presented as median and quartiles. The data with normal distribution were examined using the Shapiro–Wilk’s W test. Parameters of uncorrected visual acuity (UCVA), best-corrected visual acuity (BCVA), CCT, vertical cup-to-disc ratio, refractive error, RNFL thickness, disc area, VF MD, or VF pattern standard deviation (PSD) had skewed distributed, so a nonparametric Mann–Whitney test was used to compare these parameters. Pearson's chi-squared test was used to analyze categorical variables such as gender and TM pigmental grading. 24-h IOP characteristics (mean, peak, and fluctuation of IOP) obtained at diagnosis and different follow-up times were analyzed using a 2-way repeated-measures analysis of variance (ANOVA) with a group (SLT group and PGA group) as a between-subjects factor and different follow-up times as within-subjects factor. Greenhouse Geisser correction was applied whenever the sphericity assumption was violated. Multivariate analysis of variance process and Bonferroni correction for multiple comparisons were used to detect significant differences between groups and different measurement times for each group. A *p-*value of < 0.05 was considered statistically significant.

## Results

**Figure 1:**
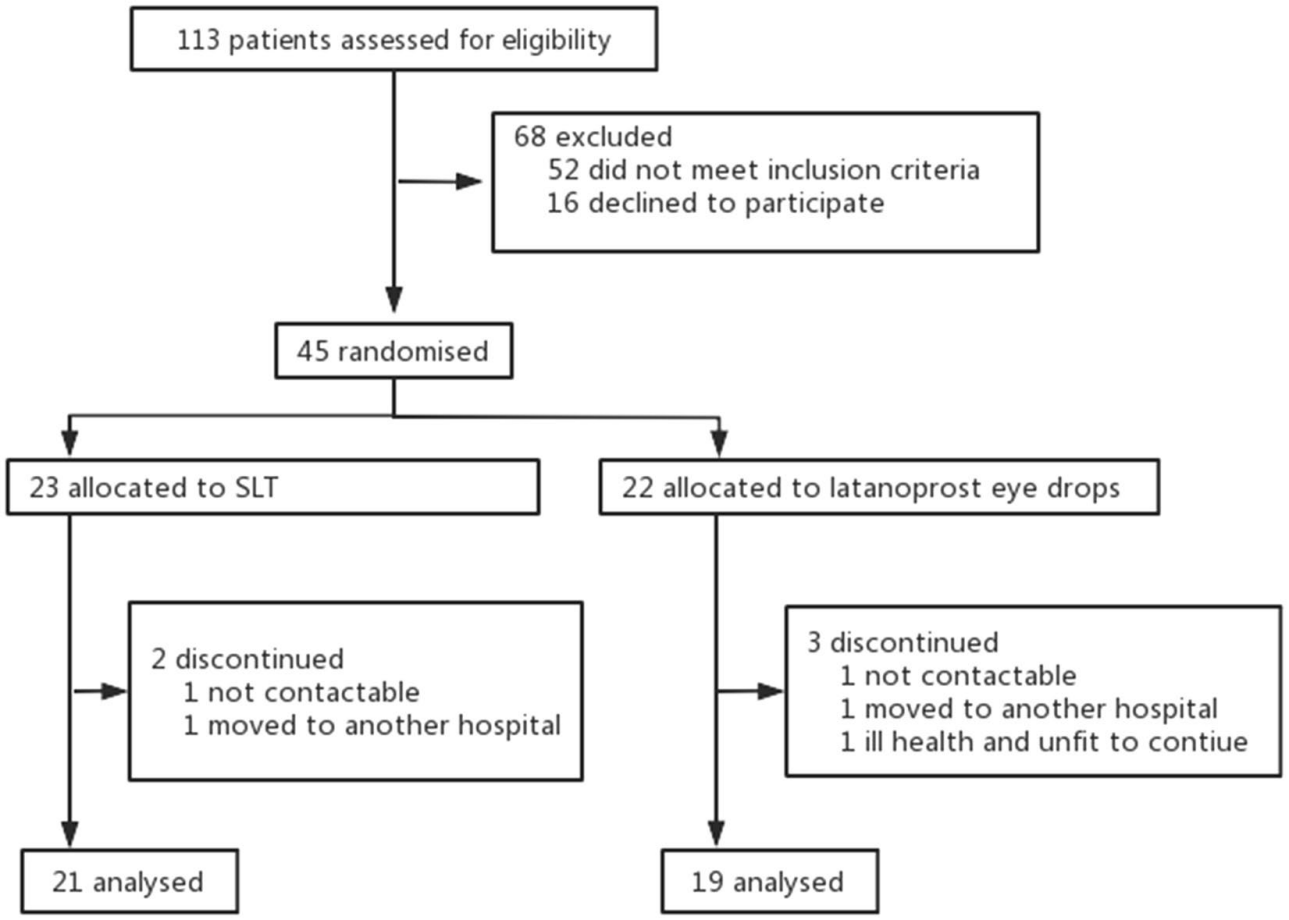
Patient including flow-chart. SLT, selective laser trabeculoplasty.

A total of 45 patients (45 eyes) were enrolled. 23 patients (23 eyes) were included in the SLT group, and 22 patients (22 eyes) were in the latanoprost group (Fig. 1). All participants were Han Chinese living in Beijing, China. At 12 weeks, 40 (88.9%) patients (40 eyes) completed follow-up, and 5 (11.1%) patients (5 eyes) dropped out. There were no significant differences in age, sex, UCVA, BCVA, diagnosis, TM pigment grading, CCT, refractive error, RNFL thickness, VF MD, and VF PSD between the two groups at baseline (Table 1). The cup-to-disc ratio measured by OCT of the latanoprost group was greater than that of the SLT group (*P* = 0.023).

**Table 1:** Participants’ demographics and ocular characteristics at baseline.

	SLT group	Latanoprost group	*P* value
Age, years, mean ± SD	47.8 ± 15.6	53.9 ± 9.9	0.098
Male/female, n (%)	13 (61.9)/8 (38.1)	13 (68.4)/6 (31.6)	0.666
UCVA, log MAR, median (IQR)	0.1 (0.0 to 0.4)	0.3 (0.0–0.9)	0.644
BCVA, log MAR, median (IQR)	0.0 (0.0–0.0)	0.0 (0.0–0.1)	0.455
POAG/OHT, n (%)	15 (71.4)/6 (28.6)	17 (89.5)/2 (10.5)	0.303
TM pigment grade 0/1/2/3, n (%)	2 (9.5) 4 (19.0)/11 (52.4)/6 (19.0)	2 (10.5) 5 (26.3)/10 (52.6)/4 (10.5)	0.864
CCT(µm), median (IQR)	550 (521–562)	535 (520–550)	0.134
Cup-to-disc ratio, median (IQR)	0.6 (0.5–0.7)	0.8 (0.6–0.8)	0.023
Refractive error (Spherical D), median (IQR)	− 0.75 (− 2.25 to 0.00)	− 0.75 (− 4.00 to 0.00)	0.859
RNFLT (µm), median (IQR)	82 (76–102)	73 (70–80)	0.062
Disc area (mm^2^), median (IQR)	2.00 (1.75–2.11)	1.92 (1.78–2.10)	0.748
Rim area (mm^2^), mean ± SD	1.11 ± 0.38	0.91 ± 0.81	0.079
VF MD (dB), median (IQR)	− 3.10 (− 5.13 to − 0.05)	− 3.10 (− 5.60 to − 2.27)	0.486
VF PSD (dB), median (IQR)	1.83 (1.67–3.60)	2.95 (1.80–5.93)	0.202

IOP, intraocular pressure; SLT, selective laser trabeculoplasty; UCVA, uncorrected visual acuity; BCVA, best-corrected visual acuity; POAG, primary open-angle glaucoma; OHT, ocular hypertension; TM, trabecular meshwork; CCT, central corneal thickness; RNFLT, retinal nerve fiber layer thickness; VF, visual field; MD, mean deviation; PSD, pattern standard deviation; dB, decibels; SD, standard deviation; IQR, interquartile range.

**Figure 2:**
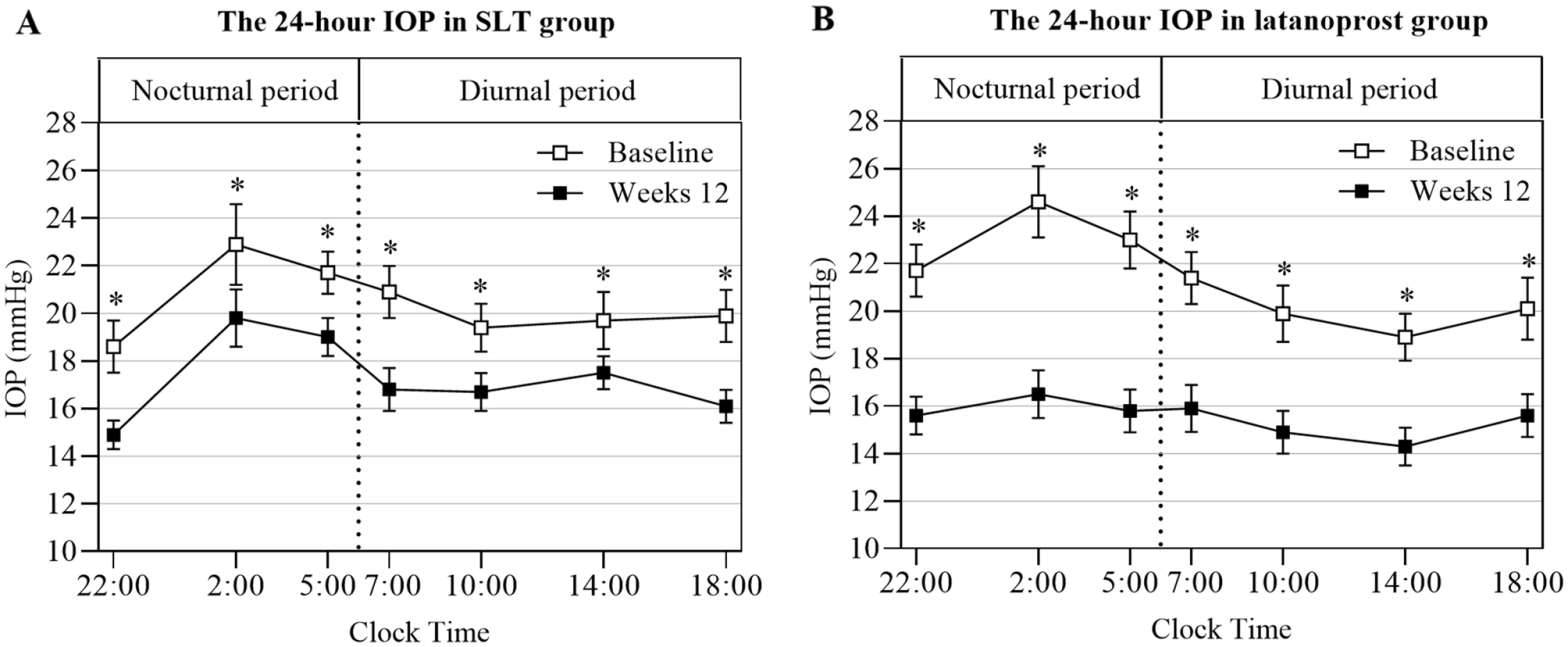
Profiles of 24-h IOP at baseline and after 12 weeks in the SLT and latanoprost groups. Measurements were taken from 21 in the SLT group (**A**) and 19 in the latanoprost group (**B**) in the seated position during the diurnal and nocturnal periods. Error bars represent the standard error of the mean. *Indicated a statistically significant difference between the baseline and week 12 (*P* < 0.05). IOP, intraocular pressure; SLT, selective laser trabeculoplasty.

Both SLT and latanoprost eye drops therapy significantly decreased IOP at 7:00, 10:00, 14:00, 18:00, 22:00, 2:00, and 5:00 (all *P*s < 0.05) when compared the baseline with the final follow-up visit (Fig. 2). Overall, there was a significant IOP reduction from baseline at each follow-up visit for each treatment group (both *P*s < 0.001). For the SLT group, the 24-h IOP reduction at weeks 1, 4, and 12 were 4.0 ± 2.8 mmHg (18.5% ± 10.4%), 2.4 ± 2.3 mmHg (11.0% ± 8.9%), and 3.2 ± 2.4 mmHg (14.3% ± 9.5%), respectively. For the latanoprost group, IOP reduction for the same periods were 5.5 ± 2.7 mmHg (24.7% ± 9.8%), 5.1 ± 2.5 mmHg (23.1% ± 9.3%), and 5.5 ± 2.0 mmHg (25.9% ± 6.8%), respectively. Therefore, IOP reduction for the latanoprost group was significantly higher than that for the SLT group at weeks 4 and 12 (*P* = 0.001 and *P* = 0.002, respectively), but not at week 1 (*P* = 0.097). The diurnal and nocturnal IOP reduction for the latanoprost group was also significantly higher than that for the SLT group at weeks 4 and 12 (all *Ps* < 0.05), but not at week 1 (both *Ps* > 0.05). At weeks 12, the IOP reduction was greater in the nocturnal periods than in the diurnal periods in the latanoprost group (6.6 ± 2.8 mmHg vs. 4.9 ± 2.2 mmHg, *P* = 0.028). In contrast, the IOP reduction was similar in the nocturnal and diurnal in the SLT group (3.2 ± 2.6 mmHg vs. 3.2 ± 3.0 mmHg, *P* = 0.952) (Table 2, Fig. 2).

**Table 2:** IOP reduction from the baseline between the SLT and latanoprost groups over time (mean ± SD, mmHg).

	Eye	1 Week	4 Weeks	12 Weeks	*P* value
24-h mean IOP reduction					
SLT group	21	4.0 ± 2.8	2.4 ± 2.3^^^	3.2 ± 2.4	0.020^†^
Latanoprost group	19	5.5 ± 2.7	5.1 ± 2.5	5.5 ± 2.0	0.088^†^
*P* value		0.097*	0.001*	0.002*	0.005^‡^
Diurnal mean IOP reduction					
SLT group	21	3.8 ± 2.8	2.4 ± 3.1^^^	3.2 ± 3.0	0.017^†^
Latanoprost group	19	5.2 ± 2.4	4.8 ± 2.6	4.9 ± 2.2^#^	0.360^†^
*P* value		0.114*	0.016*	0.048*	0.032^‡^
Nocturnal mean IOP reduction					
SLT group	21	4.4 ± 3.2	2.4 ± 2.3^^^	3.2 ± 2.6	0.009^†^
Latanoprost group	19	6.1 ± 3.6	5.9 ± 3.2	6.6 ± 2.8^#^	0.199^†^
*P* value		0.115*	0.000*	0.000*	0.002^‡^

IOP, intraocular pressure; SLT, selective laser trabeculoplasty; SD, standard deviation.

*P value: statistical significance of the difference among SLT and latanoprost groups simultaneously.

^†^*P* value: statistical significance of the difference between the time points at weeks 1, 4, and 12 within the same group.

^‡^*P* value: statistical significance of the crossover effect among 2 study groups and three measurement time points.

^^^Indicated a significant difference in mean IOP reduction between the time point and week 1 (*P* < 0.05).

^#^Indicated a significant difference when compared diurnal mean IOP reduction with nocturnal mean IOP reduction in the latanoprost group (*P* < 0.05).

**Figure 3:**
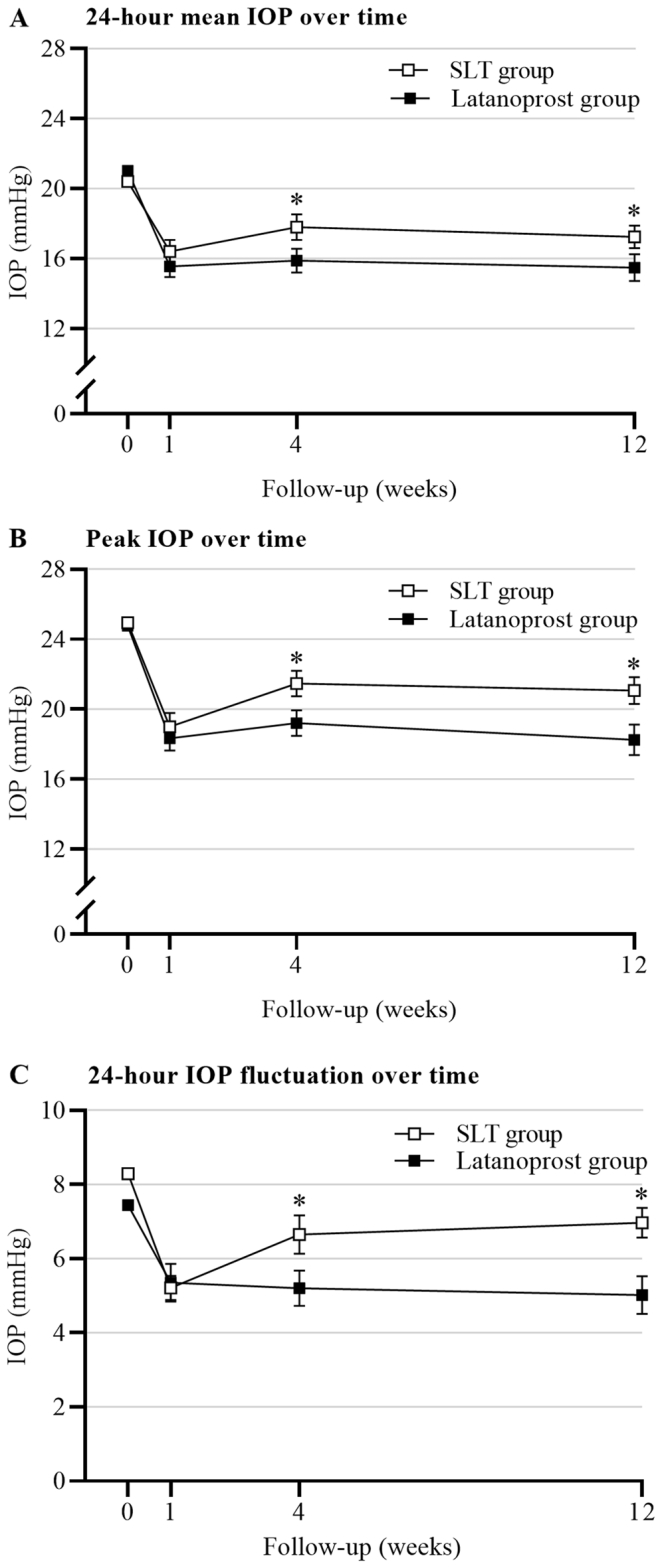
24-h IOP characteristics (mean, peak, and fluctuation of IOP) at baseline and 12-week follow-up visits. (**A**) 24-h mean IOP over time. (**B**) Peak IOP over time. (**C**) 24-h IOP fluctuation over time. *Indicated a significant difference when compared the SLT group with the latanoprost group (*P* < 0.05). IOP, intraocular pressure; SLT, selective laser trabeculoplasty.

For mean 24-h IOP, the baseline values were comparable between the SLT and the latanoprost groups (*P* = 0.668). Comparing the mean 24-h IOP between each of the follow-up visits to baseline, IOP significantly dropped at weeks 1, 4, and 12 for the SLT (all *P*s < 0.001) and the latanoprost (all *P*s < 0.001) groups. Comparing the mean 24-h IOP values between the SLT and latanoprost groups at each follow-up visit, the mean IOP was not significantly different at week 1 (*P* = 0.361), but the mean IOP was lower in the latanoprost group compared to the SLT group at weeks 4 and 12 (*P* = 0.043, and 0.026, respectively) (Table 3 and Fig. 3A).

**Table 3:** The mean 24-h IOP, peak IOP, and 24-h IOP fluctuation at each time point across 12 weeks (mean ± SD, mmHg).

	Eye	Baseline	1 Week	4 Weeks	12 Weeks	*P* value
24-Hour mean IOP						
SLT group	21	20.4 ± 4.3	16.4 ± 3.1^#^	18.0 ± 3.3^#^	17.8 ± 2.7^#^	< 0.001^†^
Latanoprost group	19	21.0 ± 4.5	15.6 ± 2.7^#^	15.9 ± 2.9^#^	15.5 ± 3.3^#^	< 0.001^†^
*P* value		0.668*	0.361*	0.043*	0.026*	0.003^‡^
Peak IOP						
SLT group	21	24.9 ± 5.2	19.0 ± 3.6^#^	21.5 ± 3.4^#^	21.1 ± 3.5^#^	< 0.001^†^
Latanoprost group	19	24.8 ± 5.0	18.3 ± 3.0^#^	19.2 ± 3.2^#^	18.3 ± 3.8^#^	< 0.001^†^
*P* value		0.912*	0.536*	0.037*	0.020*	< 0.001^‡^
24-Hour IOP fluctuation						
SLT group	21	8.3 ± 2.9	5.2 ± 1.5^#^	6.7 ± 2.4	7.0 ± 1.8	< 0.001^†^
Latanoprost group	19	7.4 ± 2.1	5.4 ± 2.2^#^	5.2 ± 1.5^#^	5.0 ± 2.2^#^	< 0.001^†^
*P* value		0.299*	0.804*	0.042*	0.004*	0.036^‡^

IOP, intraocular pressure; SLT, selective laser trabeculoplasty; SD, standard deviation.

*P value: statistical significance of the difference among SLT and latanoprost groups simultaneously.

^†^*P* value: statistical significance of the difference between the time points at baseline, weeks 1, 4, and 12 within the same group.

^‡^*P* value: statistical significance of the crossover effect among 2 study groups and 4 measurement time points.

^#^Indicated a significant difference between the time point and baseline IOP (*P* < 0.05).

The baseline peak IOP occurred at 2:00 in the SLT and the latanoprost groups, and the values were comparable between the two groups (*P* = 0.299). Compared to peak IOP at baseline, the peak IOP was significantly lower at weeks 1, 4, and 12 after treatment initiation for the SLT (all* P*s < 0.001) and the latanoprost (all* P*s < 0.001) groups. The latanoprost was significantly more effective than SLT at lowering peak IOP at weeks 4 and 12 (*P* = 0.037 and 0.020, respectively) (Table 3 and Fig. 3B).

At baseline, the 24-h IOP fluctuation was comparable between the SLT and latanoprost groups (*P* = 0.299). In the SLT group, even though 24-h IOP fluctuation showed a significant reduction from baseline at post-treatment week 1(*P* < 0.001), the value was not significantly different at weeks 4 and 12(*P* = 0.206 and 0.416, respectively) from baseline. In contrast, in the latanoprost group, 24-h IOP fluctuation showed a sustained reduction through the 12-week follow-up. The values at weeks 1, 4, and 12 were lower than the baseline (*P* = 0.024, 0.023, and 0.023, respectively). Comparing 24-h IOP fluctuation between the two groups at each follow-up visit, the values were not significantly different at week 1 (*P* = 0.804), but significantly lower in the latanoprost group compared to the SLT group at weeks 4 and 12 (*P* = 0.042, and 0.004, respectively) (Table 3 and Fig. 3C). At weeks 12, the nocturnal IOP fluctuation in the latanoprost group was lower than in the SLT group (2.8 ± 2.5 mmHg vs. 5.9 ± 2.7 mmHg, *P* = 0.001). In contrast, the diurnal IOP fluctuation was similar between the two groups (3.7 ± 1.5 mmHg vs. 3.7 ± 1.7 mmHg, *P* = 0.619).

To investigate the effectiveness of SLT and latanoprost on NTG and HTG patients, we excluded a total of 8 OHT subjects (6 from the SLT group and 2 from the latanoprost group). We then categorized POAG patients into subgroups based on HTG and NTG in each treatment group. Afterward, we compared the 24-h IOP parameters of both groups before and after interventions.

Patients with NTG experienced a significant reduction in mean 24-h IOP at all follow-up points compared to the baseline in the latanoprost group (all *P*s < 0.05). Meanwhile, in the SLT group, the mean 24-h IOP significantly decreased at weeks 1 and 4 (both *P*s < 0.05), but the value at weeks 12 did not differ significantly from the baseline (*P* > 0.05). During various visits, there was no significant difference between groups in weeks 1 and 4 (*P* = 0.370 and *P* = 0.095, respectively). However, by weeks 12, the mean IOP in the latanoprost group was significantly lower compared to the SLT group (*P* = 0.011). During the follow-up visits, it was observed that the peak value of 24-h IOP decreased significantly in the latanoprost group after treatment initiation (all *P*s < 0.05). However, there was no significant decrease in the peak value in the SLT group (all *P*s > 0.05). By the end of the follow-up visits, it was noted that the peak value in the latanoprost group was significantly lower than the SLT group (*P* = 0.022). There was no significant difference in the 24-h IOP fluctuation between the baseline and all subsequent visits after treatment initiation in both groups (all *P*s > 0.05) (Supplemental Table 1).

HTG patients experienced a notable decrease in both mean 24-h IOP and peak IOP after undergoing SLT and latanoprost treatments, with statistical significance noted at all follow-up points (all *P*s < 0.05). During weeks 1, 4, and 12, the latanoprost group showed a remarkable decrease in 24-h IOP fluctuation (all *P*s < 0.05). On the other hand, the SLT group did not exhibit any significant change in 24-h IOP fluctuation compared to baseline at weeks 4 and 12 (both *P*s > 0.05). At all time points, there were no notable differences in any variables of 24-h IOP between the two groups (all *P*s > 0.05) (Supplemental Table 2).

Following the SLT treatment, there were no significant complications reported. Of the 23 participants, 8 (34.8%) experienced minor inflammation (1 + cells) within an hour of the treatment, but none of these cases sustained inflammation during the follow-up. Four patients (17.4%) had IOP spikes, with an increase in IOP ranging from 2.4 to 5.5 mmHg. Three patients in each group received additional topical IOP-lowering drops after four weeks. One SLT case did not achieve the target IOP after adding anti-glaucomatous drugs and underwent trabeculectomy.

Due to the absence of RNFL thickness thinning and VF defect, all of the 8 OHT patients (6 in the SLT group and 2 in the latanoprost group) were excluded when analyzing the changes of RNFL thickness, VF MD, and VF PSD before and after treatment in the two groups. There were 32 POAG patients remaining, with 15 in the SLT group and 17 in the latanoprost group. After treatment, no significant changes were observed in these three parameters for both groups (all *P*s > 0.05) (Table 4).

**Table 4:** The RNFLT, VF MD, and VF PSD in POAG patients between the SLT and latanoprost groups over time, median (IQR).

	Eye	Baseline	12 Weeks	*P* value
RNFLT (µm)				
SLT group	15	77 (69–89)	77 (65–87)	0.125^†^
Latanoprost group	17	73 (69–79)	77 (69–80)	0.155^†^
*P* value		0.325*	0.644*	
VF MD (dB)				
SLT group	15	− 3.10 (− 5.43 to − 2.02)	− 3.30 (− 5.53 to − 1.96)	0.422^†^
Latanoprost group	17	− 3.83 (− 7.00 to − 2.38)	− 4.26 (− 6.13 to − 2.95)	0.826^†^
*P* value		0.770*	0.560*	
VF PSD (dB)				
SLT group	15	2.25 (1.69–4.11)	2.19 (1.73–3.49)	0.807^†^
Latanoprost group	17	3.38 (2.44–6.52)	3.64 (1.74–5.88)	0.286^†^
*P* value		0.258*	0.344*	

POAG, primary open-angle glaucoma; RNFLT, retinal nerve fiber layer thickness; VF, visual field; MD, mean deviation; PSD, pattern standard deviation; SLT, selective laser trabeculoplasty; dB, decibels; IQR, interquartile range.

*P value: statistical significance of the difference between the SLT and latanoprost groups simultaneously.

^†^*P* value: statistical significance of the difference between the baseline and weeks 12 in both groups.

## Discussion

In this randomized controlled trial, we compared the short-term efficacy of primary single SLT with 0.005% latanoprost eye drops monotherapy for controlling 24-h IOP in treatment-naïve POAG and OHT. We demonstrated that both SLT and latanoprost eye drops significantly improved 24-h IOP control, including mean, peak, and 24-h fluctuation of IOP. However, latanoprost was more effective. In addition, the nocturnal IOP-lowering efficacy of the latanoprost eye drops was better than SLT during the 12-week follow-up.

The Laser in Glaucoma and Ocular Hypertension Trial (LiGHT) study compared IOP lowering 355 OHT or OAG patients (611 eyes) who received SLT with 362 OHT or OAG patients (622 eyes) who received topical medication for two months^30^. No difference was found between topical medication and primary SLT (adjusted mean difference =  − 0.1 mmHg). In this study, SLT was less effective, and this may be due to the use of different anti-glaucoma medications as well as racial differences. Additionally, single IOP measurements were used in the LiGHT study, but 24-h IOP data was collected in the current study. We point out that single IOP measurements during office hours are insufficient to analyze the actual IOP pathology of a glaucoma patient. Many patients experience peak IOP outside of clinic hours, and peak IOPs occur at nighttime^31^.

Long-term IOP fluctuation is associated with VF progression^8^. Elevated nocturnal IOP may have a stronger impact on retinal ganglion cell loss than elevated diurnal IOP^32^. Our results showed that SLT reduced the amplitude of 24-h IOP fluctuation, but the effect lasted only one week. In contrast, the latanoprost eye drops flattened the circadian IOP curve for the 12-week follow-up period, lowering the nocturnal peak IOP better than SLT. Our results were consistent with previous studies, which showed that PGA produced stable 24-h IOP reduction^33,34^. So far, the relationship between SLT and IOP fluctuation is unclear. Lee et al.^35^ found that 24-h IOP fluctuation was significantly reduced in 18 normal tension glaucoma (NTG) patients using a contact lens sensor (CLS) IOP monitor. In contrast, Tojo and colleagues^36^ reported that the 24-h IOP fluctuation did not significantly change in 10 NTG patients treated with 360° SLT using a CLS monitoring, although nocturnal IOP fluctuation significantly decreased. Aptel et al.^37^ found that SLT did not affect the 24-h IOP pattern for 14 patients with POAG. Our data showed that the effect of SLT on circadian IOP fluctuation control lasted only one week. Post-SLT, at 4 and 12 weeks, the fluctuation amplitude returned to the level before treatment, and the nocturnal peak IOP control was lost. The explanation might be that the IOP-lowering effect of SLT fades over time^38^.

Nagar et al.^20^ compared the effect of 360° SLT with latanoprost on diurnal IOP in 40 patients with OAG and OHT. They found that latanoprost was more likely to diminish diurnal IOP fluctuation. However, they didn’t collect patients’ nocturnal IOP data. Kiddee et al.^21^ compared the effect of 360° SLT with travoprost in 58 patients with POAG and NTG during the daytime and the nighttime. They found that the lowing-IOP effect of travoprost on IOP reduction in POAG and NTG patients was significant both during the daytime and the nighttime, while the SLT's lowing-IOP effect was significant only during the nighttime rather than daytime. Our data suggested that latanoprost is more effective than SLT in controlling 24-h IOP fluctuation probably due to the attenuation effect of SLT, which is consistent with the results of previous studies^20,21^. However, it was important to note that Nagar’s study^20^ only monitored IOP during the daytime, and Kiddee’s study^21^ was followed for only 8 weeks. During our study, we monitored IOP both during the day and night, and tracked the results over a period of 12 weeks.

In Chinese patients with POAG, the percentage of those with NTG ranges from 51.43 to 83.58%^39^. When comparing the effectiveness of treatments in NTG patients (6 in the SLT group and 5 in the latanoprost group), the results showed that primary 0.005% latanoprost eye drops might be more effective in reducing the mean and peak value of 24-h IOP in NTG patients compared to single SLT. This finding might be valuable in guiding clinical practice for newly diagnosed NTG patients as it suggests that latanoprost may provide a more lasting and stable treatment option.

This study also had some limitations that warrant discussion. First, GAT measurement in the sitting position was utilized at all time points during the 24-h IOP evaluation. This may fail to represent an individual’s actual IOP variation because people usually slept lying down. Interestingly, even though lying down or taking a head-down tilt position was known to impact IOP^40^, previous studies reported that IOP was still elevated in the supine posture compared to sitting while sleeping at night^41–43^. The subjects were also awoken for IOP measurements at night, so the normal sleep–wake cycle was interrupted. Changes in diurnal physiology and hormonal levels may also influence IOP results^16^. Finally, this was a single-center study with relatively small sample size and short-term follow-up.

## Conclusion

In conclusion, as a first-line treatment, both SLT and latanoprost eye drops are effective in newly diagnosed POAG and OHT patients. The latanoprost eye drops may be better in decreasing mean and peak 24-h IOP and controlling 24-h IOP fluctuation compared to SLT.

## Supplementary Information

Supplementary Table 1.

Supplementary Table 2.

## Data Availability

The data used and/or analyzed during this study are available from the corresponding author on reasonable request.
