# Supplementary material for: 24-Hour efficacy of single primary selective laser trabeculoplasty versus latanoprost eye drops for Naïve primary open-angle glaucoma and ocular hypertension patients
Source: Sci Rep. 2023 Jul 27;13:12179. doi: 10.1038/s41598-023-38550-7 (PMC10374636; doi:10.1038/s41598-023-38550-7)
Supplement: Supplementary file 1 — Supplementary Table 1. [file 41598_2023_38550_MOESM1_ESM.docx]

**Supplemental Table 1 The mean 24-hour IOP, peak IOP, and 24-hour IOP fluctuation at each time point across 12 weeks in NTG patients (mean ± SD, mmHg).**

|  | **Eye** | **Baseline** | **1 Week** | **4 Weeks** | **12 Weeks** | ***P* Value** |
| --- | --- | --- | --- | --- | --- | --- |
| **24-hour mean IOP** |  |  |  |  |  |  |
| SLT group | 6 | 15.2±1.7 | 13.4±2.2^#^ | 14.1±1.8^#^ | 14.8±2.3 | 0.007^†^ |
| Latanoprost group | 5 | 14.0±1.2 | 12.3±1.7^#^ | 12.3±1.2^#^ | 10.9±1.5^#^ | <0.001^†^ |
| *P* Value |  | 0.160^*^ | 0.370^*^ | 0.095^*^ | 0.011^*^ | 0.013^‡^ |
| **Peak IOP** |  |  |  |  |  |  |
| SLT group | 6 | 18.1±2.1 | 16.0±2.4 | 17.6±1.5 | 18.0±2.7 | 0.039^†^ |
| Latanoprost group | 5 | 17.7±1.6 | 15.1±2.1^#^ | 16.0±2.2^#^ | 13.2±3.1^#^ | 0.007^†^ |
| *P* Value |  | 0.716^*^ | 0.530^*^ | 0.188^*^ | 0.022^*^ | 0.006^‡^ |
| **24-hour IOP fluctuation** |  |  |  |  |  |  |
| SLT group | 6 | 5.9±2.4 | 5.0±1.3 | 4.8±1.2 | 5.4±1.3 | 0.697^†^ |
| Latanoprost group | 5 | 6.5±1.7 | 5.5±2 | 6.0±2.1 | 4.0±2.7 | 0.058^†^ |
| *P* Value |  | 0.670^*^ | 0.648^*^ | 0.291^*^ | 0.273^*^ | 0.262^‡^ |

IOP, intraocular pressure; SLT, selective laser trabeculoplasty; SD, standard deviation; NTG, normal-tension glaucoma.

^*^*P* value: statistical significance of the difference among SLT and latanoprost groups simultaneously.

^†^*P* value: statistical significance of the difference between the time points at baseline, weeks 1, 4, and 12 within the same group.

^‡^*P* value: statistical significance of the crossover effect among 2 study groups and four measurement time points.

^#^ Indicated a significant difference between the time point and baseline IOP (*P* < 0.05).
