# Supplementary material for: 24-Hour efficacy of single primary selective laser trabeculoplasty versus latanoprost eye drops for Naïve primary open-angle glaucoma and ocular hypertension patients
Source: Sci Rep. 2023 Jul 27;13:12179. doi: 10.1038/s41598-023-38550-7 (PMC10374636; doi:10.1038/s41598-023-38550-7)
Supplement: Supplementary file 2 — Supplementary Table 2. [file 41598_2023_38550_MOESM2_ESM.docx]

**Supplemental Table 2 The mean 24-hour IOP, peak IOP, and 24-hour IOP fluctuation at each time point across 12 weeks in HTG patients (mean ± SD, mmHg).**

|  | **Eye** | **Baseline** | **1 Week** | **4 Weeks** | **12 Weeks** | ***P* Value** |
| --- | --- | --- | --- | --- | --- | --- |
| **24-hour mean IOP** |  |  |  |  |  |  |
| SLT group | 9 | 22.2±3.6 | 16.9±2.5^#^ | 19.0±2.6^#^ | 17.9±2.6^#^ | <0.001^†^ |
| Latanoprost group | 12 | 23.9±2.3 | 16.6±2.0^#^ | 16.9±2.3^#^ | 17.0±2.0^#^ | <0.001^†^ |
| *P* Value |  | 0.193^*^ | 0.731^*^ | 0.059^*^ | 0.374^*^ | 0.001^‡^ |
| **Peak IOP** |  |  |  |  |  |  |
| SLT group | 9 | 27.1±3.5 | 19.1±3.3^#^ | 22.6±3.1^#^ | 21.0±3.2^#^ | <0.001^†^ |
| Latanoprost group | 12 | 27.3±2.2 | 19.4±2.6^#^ | 20.0±2.7^#^ | 19.9±2.1^#^ | <0.001^†^ |
| *P* Value |  | 0.878^*^ | 0.821^*^ | 0.053^*^ | 0.351^*^ | 0.103^‡^ |
| **24-hour IOP fluctuation** |  |  |  |  |  |  |
| SLT group | 9 | 8.8±2.2 | 5.1±1.7^#^ | 7.1±3 | 6.6±1.5 | 0.018^†^ |
| Latanoprost group | 12 | 7.7±2 | 5.1±2.3^#^ | 5.5±1.7^#^ | 5.4±2.1^#^ | 0.003^†^ |
| *P* Value |  | 0.251^*^ | 0.990^*^ | 0.143^*^ | 0.136^*^ | 0.601^‡^ |

IOP, intraocular pressure; SLT, selective laser trabeculoplasty; SD, standard deviation; HTG, high-tension glaucoma.

^*^*P* value: statistical significance of the difference among SLT and latanoprost groups simultaneously.

^†^*P* value: statistical significance of the difference between the time points at baseline, weeks 1, 4, and 12 within the same group.

^‡^*P* value: statistical significance of the crossover effect among 2 study groups and four measurement time points.

^#^ Indicated a significant difference between the time point and baseline IOP (*P* < 0.05).
